# Isolated ectopia lentis with partial anterior dislocation and pupillary block: a case report

**DOI:** 10.1186/s12886-024-03455-0

**Published:** 2024-04-25

**Authors:** Ariel Chen, Angela M. Ngo, Michael X. Repka, Courtney L. Kraus

**Affiliations:** 1 Northwest Permanente, Portland, OR USA; 2 Georgetown University School of Medicine, Washington, DC USA; 3 Wilmer Eye Institute, Johns Hopkins University School Medicine, Baltimore, MD USA

**Keywords:** Pediatric ectopia lentis, Pupillary block, Isolated ectopia lentis

## Abstract

**Background:**

Ectopia lentis is the dislocation of the natural crystalline lens and usually presents in the setting of trauma or other systemic diseases. Herein, we describe a case of an otherwise healthy four-year-old boy with isolated ectopia lentis whose partial lens dislocation was captured on a smartphone by the patient’s father several days prior.

**Case presentation:**

A four-year-old boy with no past medical, developmental, or trauma history presented with bilateral partial anterior lens dislocation with pupillary block. Initial ophthalmic evaluation two months prior was notable for uncorrected visual acuity at 20/100 OD, 20/250 OS, bilateral iridodenesis, and partially dislocated lenses inferonasally OD and inferiorly OS on slit lamp. Genetic testing found no abnormalities. Ten months later, the patient developed sudden onset of left eye pain. A dislocated lens and temporarily dilated left pupil were captured on a smartphone by the patient’s father. He was evaluated 3 days later after a second episode and found to have hand motion vision OS, a fixed 8 mm left pupil with the crystalline lens subluxed into the pupil space and accompanying intraocular pressure OS of 40 mmHg. The lens was surgically removed with a limited anterior vitrectomy. Four and a half years after surgery, visual acuity was 20/125 OS with aphakic correction. The right eye eventually underwent prophylactic lensectomy and was 20/30 in aphakic correction.

**Conclusions:**

This report presents a unique presentation of isolated ectopia lentis with anterior lens dislocation and pupillary block and illustrates the role of smartphone photography in assisting in the triage of eye emergencies.

## Background

Ectopia lentis is the dislocation or displacement of the natural crystalline lens. It is associated with systemic syndromes or due to secondary causes such as trauma but can also be seen in isolation [1] Complications of ectopia lentis include amblyopia in children, cataracts, posterior or anterior dislocation, and lens-induced glaucoma or uveitis [2]. In this report, we describe a case of ectopia lentis complicated by partial anterior lens dislocation with pupillary block.

## Case presentation

A healthy, four-year-old boy was referred to the Division of Pediatric Ophthalmology at the Wilmer Eye Institute, Baltimore, Maryland for bilateral partial lens dislocation from an outside ophthalmologist. Two months prior, the patient had failed a vision screening at school and was referred for further evaluation. The child denied any visual symptoms; however, the family noticed that he would sit very close to the television and zone in closely on toys. He had no history of trauma, developmental issues, or hearing deficits. He had a normal genetic screening at birth. Multigenerational family history was negative for any ocular problems other than myopia in his mother.

On initial ophthalmic examination, uncorrected visual acuity was 20/100 in the right eye and 20/250 in the left eye. Pupils were equally round and reactive to light and intraocular pressure was normal by palpation. The child had no measurable stereopsis and suppression of his left eye on Worth 4-dot test. He was orthophoric at all distances. Slit-lamp examination was notable for iridodenesis in both eyes and partially dislocated lenses; inferonasally in the right eye and inferiorly in the left eye. Cycloplegic refractive error was − 14.50DS + 3.75DC x 005° in the right eye and − 19.00 DS + 6.00 DC x 065°.During a later examination under anesthesia, it was noted that the macula OU were within normal limits. Retinal vessels were normal without attenuation OU. Peripheral exam was unremarkable OU. Pachymetry measurements were 575 microns on the right eye and 584 microns the left eye. Axial length was 21.83 mm in the right eye and 21.12 mm in the left eye. He was prescribed glasses and referred for a genetics evaluation.

General physical examination found no evidence of any musculoskeletal features of Marfan syndrome or homocystinuria. Echocardiography revealed normal aortic dimensions. Genetic testing showed negative sequencing for deletion or duplication of fibrillin-1 (FBN-1) and cystathionine beta-synthase (CBS).

**Fig. 1:**
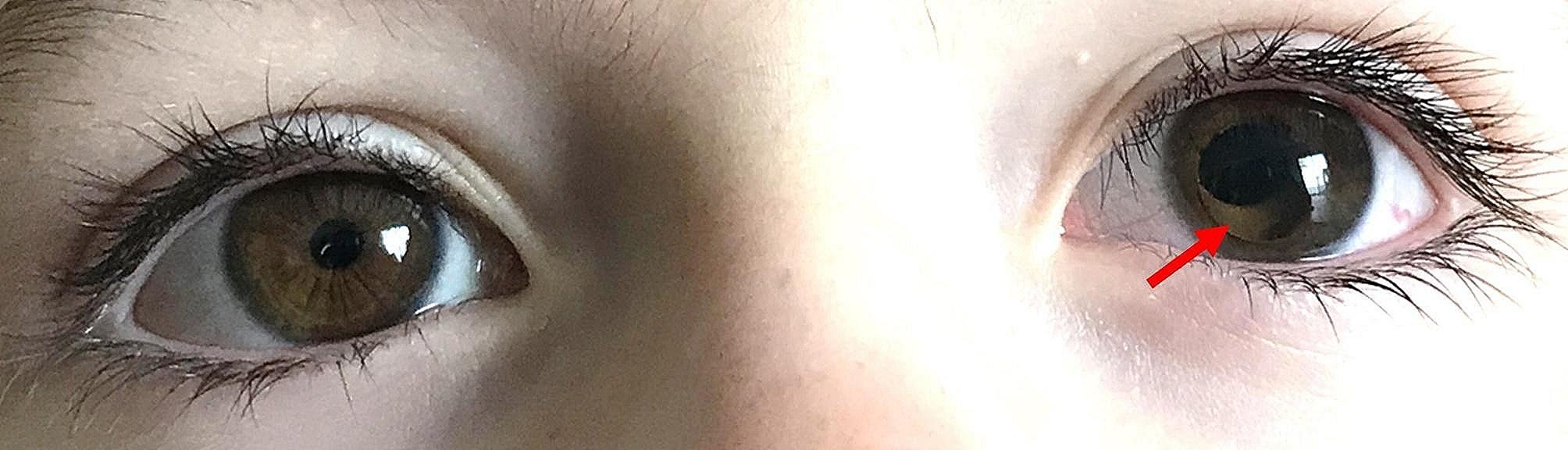
Partial lens dislocation and pupillary block of the left eye captured on smartphone. The patient’s partial lens dislocation and pupillary block of the left eye was captured on smartphone by the patient’s father. The patient had developed sudden onset left eye pain and was evaluated several days later after a second similar episode. This case of ectopia lentis is rare in occurring in an otherwise healthy patient with no medical, developmental, or trauma history

Ten months after initial presentation, the patient developed sudden onset of left eye pain and nausea that lasted for approximately an hour. During the episode, his parents noticed that the pupil of the left eye was temporarily dilated, as seen in Fig. 1, an image captured by the father. Three days later, a second episode occurred, at which time he was evaluated and found to have decreased vision in the left eye to hand motion, a fixed, 8 mm pupil, and an intraocular pressure of 40 mmHg by applanation. Slit lamp examination revealed partial anterior lens dislocation with pupillary block. Patient was placed in a supine position and given hydroxyamphetamine hydrobromide and tropicamide 1% with no resolution of symptoms. A laser peripheral iridotomy was unsuccessfully attempted.

Later that day, he was taken to the operating room. The lens was observed to be of normal size and prolapsed into the pupil space creating pupillary block. A 30-gauge needle with balanced salt solution was used to deepen the chamber followed by injection of Healon into the anterior chamber. Using a 25- gauge vitrector, the lens was removed, followed by a limited anterior vitrectomy. The child was left aphakic.

On post-operative day one, intraocular pressure was 17 mmHg in the left eye with resolution of pain and nausea. Five months after surgery, best-corrected visual acuity was 20/250 in the left eye and intraocular pressure was 12 mmHg. The cornea was clear. The right eye had no similar event and was treated with daily administration of pilocarpine 1% until prophylactic lensectomy two years later. Four and a half years after the initial OS surgery, visual acuity was 20/125 in the left eye with aphakic correction and 20/30 in the right eye with aphakic correction.

## Discussion and conclusions

Ectopia lentis occurs when the zonular filaments have compromised structural integrity due to intrinsic or extrinsic factors. Systemic associations of ectopia lentis include Marfan syndrome, homocystinuria, Weill-Marchesani syndrome, Ehlers-Danlos syndrome, sulfite oxidase deficiency and hyperlysinemia. Ectopia lentis may also occur secondary to trauma, anterior uveal tumors, pseudoexfoliation, hypermature cataracts, and macrophthalmia [1]. Cases of isolated ectopia lentis with no systemic comorbidities or secondary associations do occur. Due to the weakened zonular filaments, the less secured lens may take on a rounded shape, leading to high myopia that is often associated with isolated ectopia lentis [3]. Differentiating syndromic versus isolated ectopia lentis is important to guide clinical management and surveillance of young patients. Recently, genetic mutations to ADAMTSL4, LTPB2, and FBN-1 have been associated with isolated ectopia lentis [2–4].

Our patient was a high myope bilaterally, which may predispose to lens sublocation in both eyes, though notably he was negative for mutations in FBN-1 and CBS, which are associated with Marfan syndrome and homocystinuria respectively. These two conditions with associated ectopia lentis have important systemic associations that confer significant cardiac risk to an affected individual. Further genetic evaluation was not pursued for our patient. Currently, there are no guidelines for ongoing medical evaluations of patients with isolated ectopia lentis. For this case, annual cardiac echography was recommended by the genetics department.

Complications of ectopia lentis include amblyopia in children, posterior or anterior dislocation of the lens, cataracts, and lens-induced glaucoma or uveitis. Anterior dislocation of the lens can induce secondary glaucoma, uveitis, and corneal endothelial damage requiring urgent intervention [5–7]. 

Similar to our patient, case reports of patients with anteriorly dislocated lens were most commonly treated surgically with lens extraction and anterior vitrectomy [5, 6, 8–11]. Of these patients, many had reported scleral fixated intraocular lens placed intraoperatively [5, 9, 10] or as a separate surgery a few months later [6]. It is also reasonable to leave pediatric patients aphakic and manage with aphakic glasses or contact lenses, given the absence of capsular support [11, 12]. Alternative management to surgery does have reported success. A case reported by Garza-Leon and de la Para-Colin treated a completely anteriorly dislocated lens with supine positioning, tropicamide, and phenylephrine [7]. Few of these cases reported decreased endothelial cell count post-operatively [5, 7, 8, 10]; otherwise, patients recovered with good post-operative visual outcomes and intraocular pressures.

In our case, his visual acuity did not improve as much as anticipated. This may be due to a combination of amblyopia from his anisometropia found on initial exam, or injury to the optic nerve or retina from elevated intraocular pressures. As for his right eye, initially his right eye had minimal dislocation and no elevation of intraocular pressure, therefore management was daily pilocarpine and observation. However over time, due to concern for worsening lens position, the right eye subsequently underwent prophylactic intervention with lensectomy and anterior vitrectomy, after which he has done quite well in aphakic correction.

## Data Availability

No datasets were generated or analysed during the current study.

## Abbreviations

OD: Oculus Dexter, Right Eye
OS: Oculus Sinister, Left Eye
FBN-1: Fibrillin gene
CBS: cystathionine beta-synthase gene

## References

[1] Sadiq MA, Vanderveen D (2013). Genetics of Ectopia Lentis. Semin Ophthalmol.

[2] Esfandiari H, Ansari S, Mohammad-Rabei H, Mets M. Management strategies of ocular abnormalities in patients with marfan syndrome: current perspective. J Ophthalmic Vis Res. 2019;14(1). 10.4103/jovr.jovr_29_18 PMC6388525 30820290

[3] Neuhann TM, Stegerer A, Riess A, Blair E, Martin T, Wieser S et al. ADAMTSL4-associated isolated ectopia lentis: further patients, novel mutations and a detailed phenotype description. Am J Med Genet A. 2015;167(10). 10.1002/ajmg.a.37157 25975359

[4] Chandra A, Aragon-Martin JA, Hughes K, Gati S, Ashwin Reddy M, Deshpande C et al. A genotype-phenotype comparison of ADAMTSL4 and FBN1 in isolated ectopia lentis. Invest Ophthalmol Vis Sci. 2012;53(8). 10.1167/iovs.12-9874 22736615

[5] Kim YJ, Ha SJ. Intracapsular lens extraction for the treatment of pupillary block glaucoma associated with anterior subluxation of the crystalline lens. Case Rep Ophthalmol. 2013;4(3). 10.1159/000356530 PMC3861857 24348413

[6] Choi DY, Kim JG, Song BJ. Surgical management of crystalline lens dislocation into the anterior chamber with corneal touch and secondary glaucoma. J Cataract Refract Surg. 2004;30(3). 10.1016/j.jcrs.2003.07.012 15050275

[7] Garza-Leon M, De La Parra-Colín P. Medical treatment of crystalline lens dislocation into the anterior chamber in a patient with Marfan syndrome. Int Ophthalmol. 2012;32(6). 10.1007/s10792-012-9592-7 22692227

[8] Kawashima M, Kawakita T, Shimazaki J. Complete spontaneous crystalline lens dislocation into the anterior chamber with severe corneal endothelial cell loss. Cornea. 2007;26(4). 10.1097/ICO.0b013e3180303ae7 17457202

[9] Kwon YA, Bae SH, Sohn YH. Bilateral spontaneous anterior lens dislocation in a retinitis pigmentosa patient. Korean J Ophthalmol. 2007;21(2). 10.3341/kjo.2007.21.2.124 PMC2629702 17592245

[10] Seong M, Kim MJ, Tchah H. Argon laser iridotomy as a possible cause of anterior dislocation of a crystalline lens. J Cataract Refract Surg. 2009;35(1). 10.1016/j.jcrs.2008.07.035 19101444

[11] Vasavada AR, Praveen MR, Desai C. Management of bilateral anterior dislocation of a lens in a child with Marfan’s syndrome. J Cataract Refract Surg. 2003;29(3). 10.1016/s0886-3350(02)01529-8 12663033

[12] Mukhopadhyay U, Chakrabort C, Mondal A, Pattyanayak U, Agarwal R, Tripathi P. Spontaneous dislocation of a crystalline lens to the anterior chamber with pupillary block glaucoma in Noonan Syndrome: a case report. Pan Afr Med J. 2014;17. 10.11604/pamj.2014.17.135.3049 PMC4218645 25374640

